# Ball-Milling-Assisted Coating and Magnetic Properties of Fluorescent Biodegradable Powders for Fingerprint Detection

**DOI:** 10.3390/molecules30224481

**Published:** 2025-11-20

**Authors:** Hélio L. Barros, Nuno Martinho, Susana Cardoso, Vasco D. B. Bonifácio

**Affiliations:** 1 iBB-Institute for Bioengineering and Biosciences and i4HB-Institute for Health and Bioeconomy, Instituto Superior Técnico, Universidade de Lisboa, Av. Rovisco Pais, 1049-001 Lisboa, Portugalvasco.bonifacio@tecnico.ulisboa.pt (V.D.B.B.); 2 Instituto Superior Técnico, Universidade de Lisboa, Av. Rovisco Pais, 1049-001 Lisboa, Portugal; 3 Instituto de Engenharia de Sistemas E Computadores para Microsistemas e Nanotecnologias (INESC MN), Rua Alves Redol, 9, 1000-029 Lisboa, Portugal

**Keywords:** fingermarks, mechanochemistry, multifunctional powders, forensics

## Abstract

The development of environmentally friendly materials for forensic applications is a growing area of interest. Traditional forensic methods often rely on resource-intensive processes and hazardous materials, and thus a demand for sustainable efficient materials without compromising performance is needed. Fluorescent, regular and magnetic powders were prepared through ball-milling-assisted coating using biodegradable matrices such as silica, chitosan, and tri-sodium citrate. The effect of the magnetic core on the optical properties, along with the influence of matrix type on the photophysical and magnetic properties of the powders, was assessed. The results suggest that the polymeric matrix effectively prevented fluorescence quenching, although a reduction in fluorescence intensity was observed when comparing magnetic and non-magnetic powders. For core–shell structures based on chitosan and tri-sodium citrate, the reduction in fluorescence caused by the absorption of the magnetic core (Fe_3_O_4_) was less pronounced. Additionally, these structures exhibited better magnetic properties when compared with a silica-based core–shell. All fluorescent powders proved highly efficient in fingerprint detection on various surfaces, yielding similar results to commercially available powders. The produced powders are not only safe and cost-effective but also environmentally friendly, making them an alternative to the current commercial powders used in forensic applications.

## 1. Introduction

The field of forensic science continually seeks innovative methods to enhance the efficiency and precision of fingerprint detection, a critical component in criminal investigations. Among the various techniques used to develop and visualize latent fingerprints, fluorescent powders have gained significant attention due to their high sensitivity and versatility [1,2]. However, regular powders often rely on hazardous chemicals and non-degradable matrices, raising environmental concerns [3,4]. These challenges highlight the need for alternative solutions rooted in green chemistry principles, aiming to reduce the ecological footprint of forensic practices.

Recent advancements have explored biodegradable materials as potential carriers for fingerprint powders, offering a sustainable approach to forensic evidence collection [5,6,7]. In particular, the integration of biodegradable matrices with fluorescent and magnetic properties presents a promising avenue for enhancing both the detection and visualization of latent fingerprints [8]. Despite these strides, limited research has systematically examined the intersection of optical and magnetic characteristics in biodegradable materials tailored for fingerprint detection.

Organic-based molecules play a vital role in forensic detection due to their delicate balance between hydrophobic and hydrophilic groups, solid-state fluorescence emission, and suitability for large-scale production [9,10]. Particularly, for fingerprint development on multicolored surfaces, fluorescent powders with the ability to absorb specific wavelengths of light and emit light at different wavelengths based on surface hydrophobicity are crucial [11,12].

Among fluorophores, coumarins stand out due to their remarkable fluorescence properties, including high photostability, quantum yield, absorption coefficient, ease of modification, and large Stokes shift [13,14]. In recent years, coumarin derivatives have been extensively applied in various fields, such as metal ion sensors [15,16], organic light-emitting diodes (OLEDs) [17], fluorescent probes [18], oxidase inhibitors [19], protein labeling [20] and pH probes [21].

Researchers have also explored the incorporation of different fluorophores into various matrices for forensic applications, demonstrating significant advancements in fingerprint visualization [22,23,24,25]. For instance, Ding et al. [26] and Pan et al. [27] developed bifunctional composite powders combining moderate magnetic properties with intense fluorescence properties, highlighting the potential of multifunctional powders in forensic science. However, these studies often overlook critical real-world challenges such as toxicity, safety, and environmental sustainability.

This work focuses on the design, ball-milling-assisted coating and characterization of multifunctional powders based on biodegradable matrices and a coumarin fluorophore, with particular emphasis on their optical and magnetic properties. By employing a green chemistry approach, this research aims to contribute to both the sustainability and efficacy of fingerprint detection methods. Specifically, we examined regular and magnetic fluorescent powders based on tri-sodium citrate, chitosan and silica matrices and their capacity to produce clear and durable fingerprint impressions on various types of surfaces. The main goals include evaluating the effect of different polymeric matrices on fluorescent intensity, magnetic responsiveness, and overall performance compared to commercial powders, and highlighting their potential as viable effective alternatives for forensic applications.

## 2. Results and Discussion

### 2.1. Ball-Milling-Assisted Coating of Fluorescent Magnetic Powders

**Figure 1:**
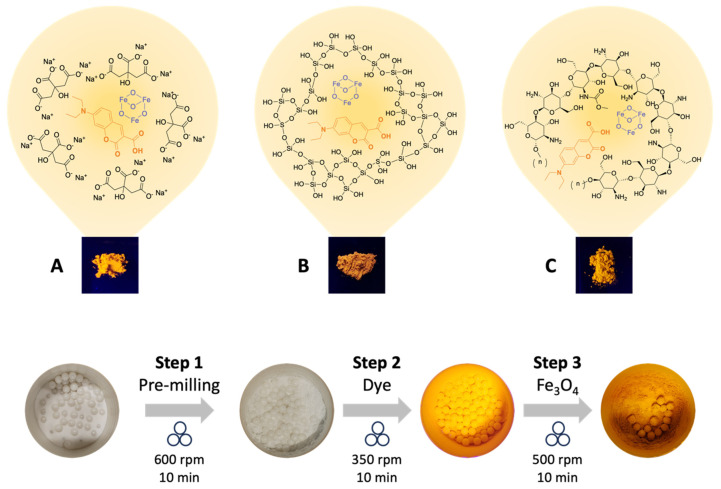
Ball-milling-assisted coating of fluorescent magnetic powders based on tri-sodium citrate (**A**), silica (**B**) and chitosan (**C**).

The process involved three sequential steps. Initially, the matrix underwent pre-treatment in a planetary ball mill to decrease its particle size. Subsequently, the regular fluorescent powders (DCC@CIT, DCC@CHT and DCC@SIL) were obtained by incorporation of dye on matrices (1:3) in a planetary ball mill. Finally, the magnetic fluorescent powders (m-DCC@CIT, m-DCC@CHT and m-DCC@SIL) were synthesized by adding Fe_3_O_4_ (40:1) (Figure 1). The dye:matrix:Fe_3_O_4_ (10:30:1) ratio was optimized, and the reduction in the number of balls in the last step, while increasing the rotation speed during the process, were found as key conditions to prevent fluorescence quenching caused by the magnetic core (Fe_3_O_4_).

### 2.2. Optical Properties of Fluorescent Magnetic Powders

**Figure 2:**
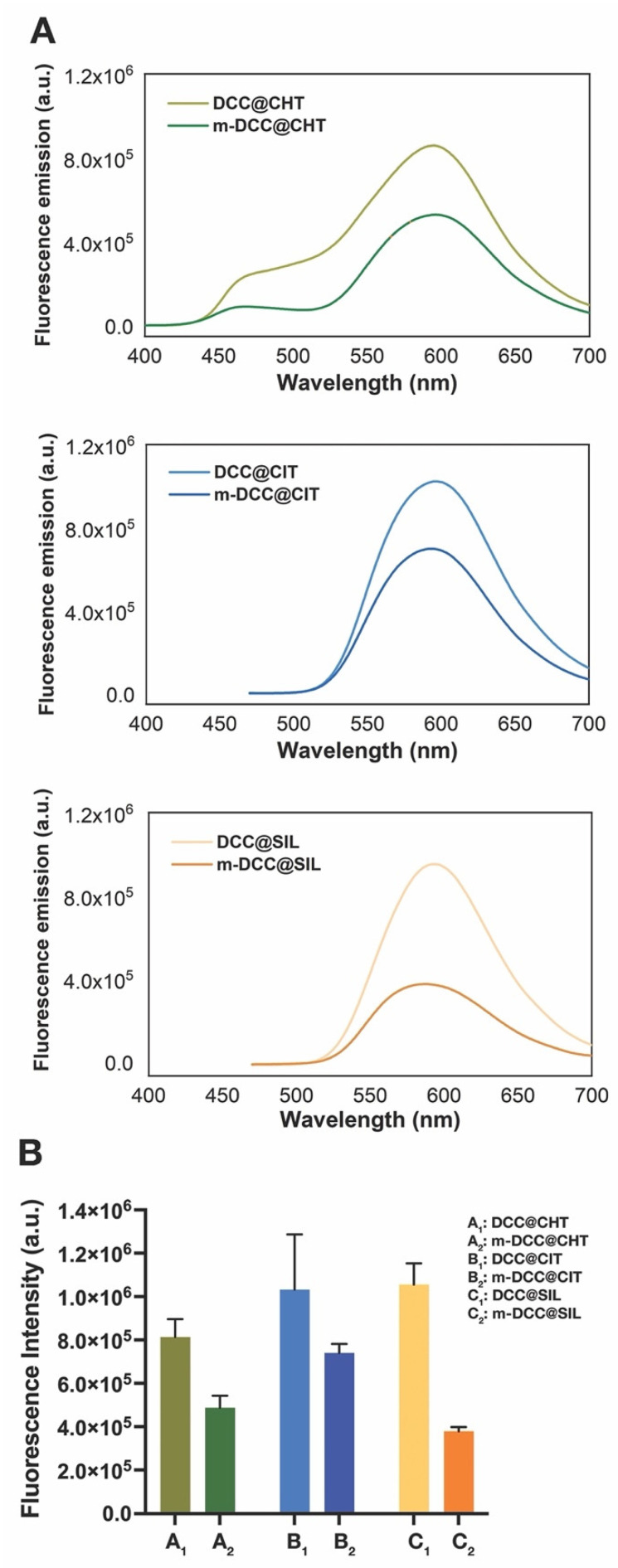
Fluorescence spectra of regular and magnetic powders (**A**) based on chitosan, tri-sodium citrate, and silica under UV light (λ = 365 nm). Bar graph (**B**) showing the maximum fluorescence intensity of regular and magnetic powders.

Photophysical stability and strong solid-state emission are critical features for the effective use of fluorescent powders in latent fingerprint detection. As shown in Figure 2, the emission spectra of both regular and magnetic fluorescent powders indicate that the matrix type had no significant effect on the maximum emission wavelengths. The powders exhibited maximum emission wavelengths between 520 and 700 nm with excitation at 365 nm. For chitosan-based powders, an additional emission band was observed around 460 nm, which corresponds to chitosan autofluorescence.

Our data aligns with previous findings [17], where the dye incorporation into matrices was found to occur primarily through electrostatic interactions and hydrogen bonding, which did not alter the intrinsic dye emission.

However, a substantial reduction in fluorescence intensity was observed when comparing magnetic and non-magnetic powders. This suggests that the magnetic core (Fe_3_O_4_) influences the photophysical properties, likely due to partial fluorescence quenching by the magnetic core. The fluorescence quenching in magnetic powders is a known challenge, as the iron oxide core can absorb emitted light [10]. Although the magnetic core reduced emission intensity, its fluorescence remained strong enough to support its application in forensic settings.

In core–shell structures, particularly those using tri-sodium citrate, the reduction in fluorescence intensity was significantly less pronounced. This suggests that these matrices provide higher degree of protection to the dye in comparison with chitosan and silica, minimizing the quenching effect.

### 2.3. Magnetic Properties of Fluorescent Magnetic Powders

**Figure 3:**
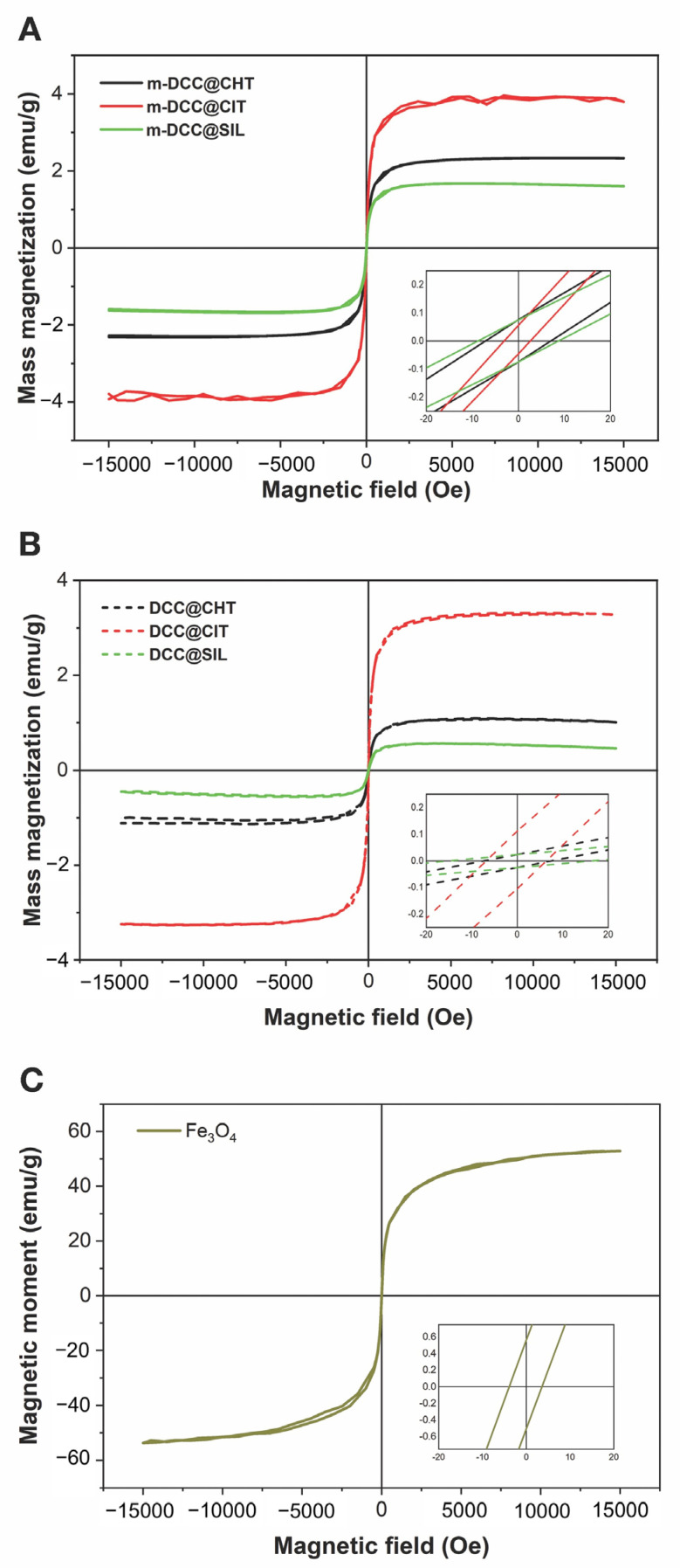
Magnetization curves vs. applied magnetic field of synthesized magnetic matrices (**A**), magnetic fluorescent powders (**B**) and Fe_3_O_4_ (**C**). Inset: magnified view of the central region of the curve.

The powders were magnetically characterized to assess the effect of the matrix on their magnetization properties. Vibrating sample magnetometry (VSM) was used to determine the saturation magnetization, coercivity, and remanent magnetization of the samples. Figure 3 presents the magnetization curves as a function of the magnetic field for magnetite (Fe_3_O_4_), non-fluorescent magnetic powders (m-CHT, m-CIT, and m-SIL) and the fluorescent magnetic powders (m-CIT, m-CHT and m-SIL) and the fluorescent magnetic powders (m-DCC@CIT, m-DCC@CHT and m-DCC@SIL).

The saturation magnetization (Ms), remanent magnetization (Mr), and coercivity (Hc) values are summarized in Table 1. The Ms values are consistent with those reported in the literature for magnetite synthesized via coprecipitation in an alkaline medium (52–78 emu/g) [28,29,30]. The absence of a hysteresis loop, along with the very low values of remanent magnetization and coercivity, indicates that the powder exhibits superparamagnetic behavior. This means that while the particles are strongly attracted to a magnetic field, their magnetization returns to zero once the field is removed [30].

When comparing the magnetization curves of Fe_3_O_4_ with polymer coatings (A and B) to those without coatings (C) (Figure 3), it is evident that the material coating significantly impacts the magnetic behavior of the powders. Although powders with different coatings also exhibited superparamagnetic behavior, their Mr values were lower than those of the uncoated powders. This result aligns with previous studies [31], which observed Ms reduction in magnetite samples coated with silica and their subsequent functionalization. This behavior is expected since the Mr is measured per unit weight of the sample, and not just considering the magnetite core. The diamagnetic layer on the surface, formed by the matrix, also contributes to Mr reduction [32,33].

Despite the very low Hs values, a slight increase was observed in the coated powders. Furthermore, the fluorescent magnetic powders demonstrated higher Ms and Mr values and lower Hs values compared to their non-fluorescent counterparts. These differences may be related to the chemical functionalization of the magnetic core with organic molecules, which interact with the coatings [32].

### 2.4. Infrared Spectra of Fluorescent Magnetic Powders

**Figure 4:**
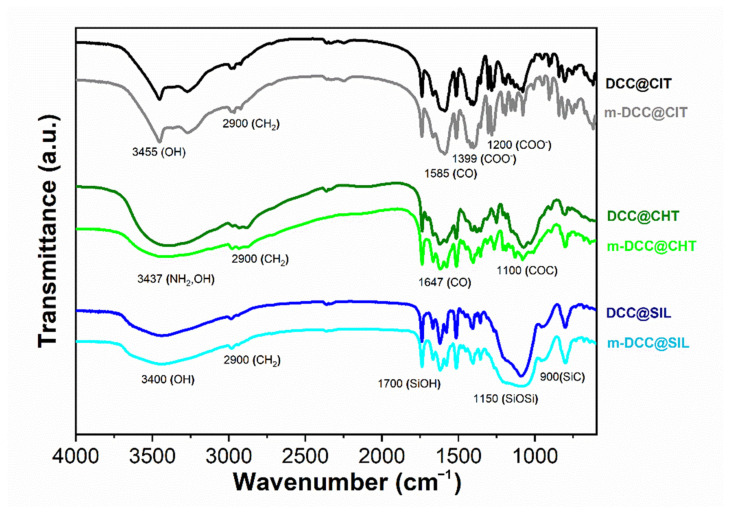
FTIR spectra of regular (DCC@CIT, DCC@CHT and DCC@SIL) and magnetic powders (m-DCC@CIT, m-DCC@CHT and m-DCC@SIL).

The infrared spectra of fluorescent magnetic powders based on tri-sodium citrate, chitosan, and silica are presented in Figure 4. All powders showed characteristic bands at 3400 cm^−1^ (OH and NH_2_ stretching) and 2900 cm^−1^ (C-H stretching). For tri-sodium citrate-based powders, in addition to these bands, the spectra also showed bands at 1580 cm^−1^ (C=O stretching), 1399 cm^−1^ (C–H bending), and 1200 cm^−1^ (C-O stretching). In the chitosan-based powders, additional bands were observed at 1647 cm^−1^ (attributed to C=O vibration of acetylated units, amide I) and 1100 cm^−1^ (C-O stretching). The silica-based powders exhibited typical bands at 1700 cm^−1^ (SiOH stretching), 1150 cm^−1^ (SiOSi stretching), and 900 cm^−1^ (SiC stretching).

The spectra of the fluorescent regular powders (non-magnetic) and fluorescent magnetic powders showed primarily matrix-related bands. The characteristic bands of the dye and magnetite were not clearly perceptible, either due to overlap, as they share similar functional groups with the matrix, or due to the significant difference in proportions. The synthesized powders revealed similar band profiles with identical wavenumbers to those of the isolated matrices [34,35,36], suggesting no chemical reaction occurred between the compounds present in the powder’s formulation. These results indicate a predominance of physical interactions in the encapsulation process, consistent with previous studies involving other dyes and matrices [21,37,38].

**Figure 5:**
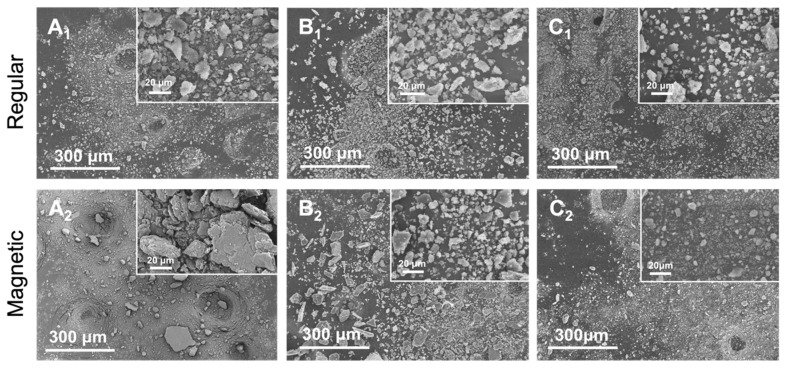
SEM images of regular ((**A_1_**): DCC@CHT, (**B_1_**): DCC@CIT, (**C_1_**): DCC@SIL) and magnetic fluorescent powders ((**A_2_**): m-DCC@CHT, (**B_2_**): m-DCC@CIT, (**C_2_**): m-DCC@SIL).

The morphological characterization of the powders was conducted using Scanning Electron Microscopy (SEM). SEM analysis revealed that both regular and magnetic powders displayed particle sizes mostly ranging from 4 to 30 µm, although agglomeration of particles was also observed (Figure 5). Both regular and magnetic powders exhibited irregular sizes and shapes. The irregular shapes and size distribution observed are consistent with our previous studies and literature studies [39], indicating that the milling process does not produce uniformly sized particles, but rather a heterogeneous mixture.

### 2.5. Detection of Fingerprint on Different Type of Surfaces

**Figure 6:**
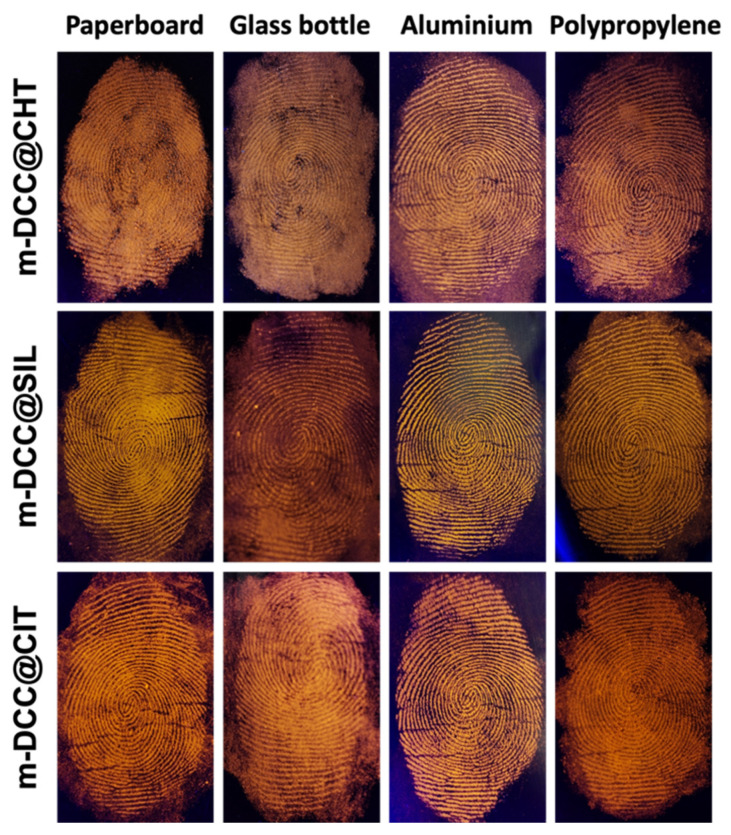
Image of fingerprint detected with fluorescent magnetic powders on different types of surfaces (paperboard, glass bottle, aluminum and polypropylene) under UV light (λ = 365 nm).

Figure 6 shows images of fingerprints detected on various surfaces using magnetic powders under UV light at 365 nm. As observed, powders demonstrated high efficiency, selectively adhering to fingerprint residues. All powders emit intense orange fluorescence, with slight variations in tone, allowing for perfect capture of fingerprints.

### 2.6. Sensibility Studies by Sequential Deposition

**Figure 7:**
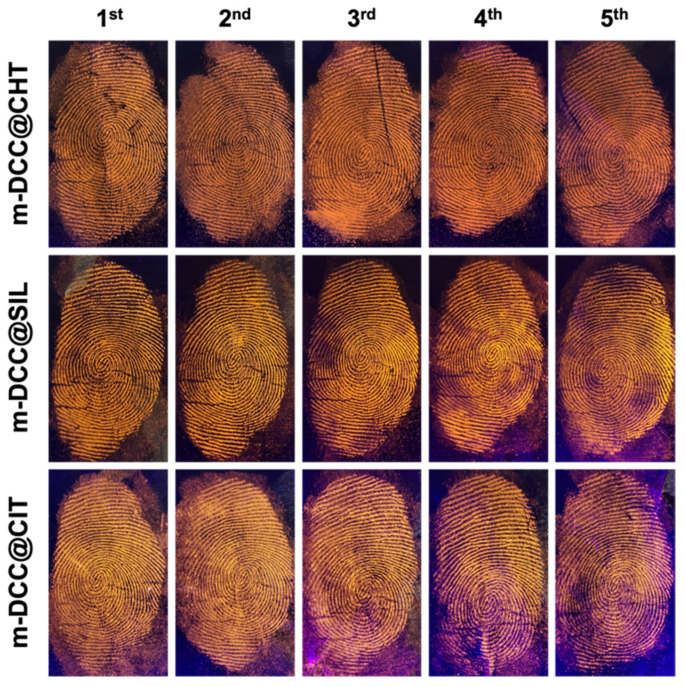
Images of fingerprint deposited in sequence (1–5) on glass surfaces and detected with fluorescent magnetic powders under UV light (λ = 365 nm).

The sensitivity of the fluorescent magnetic powders was evaluated through sequential fingerprint deposition. The approach involved depositing fingerprints sequentially on aluminum surfaces, without any contact or recharging between the depositions, resulting in a series of fingerprints with progressively decreasing amounts of residue. All developers produced similar results (Figure 7), allowing the visualization of fingerprints up to the fifth sequential deposition, demonstrating high performance even with low residue concentrations.

### 2.7. Comparison Between Regular and Magnetic Fingerprint Developers

**Figure 8:**
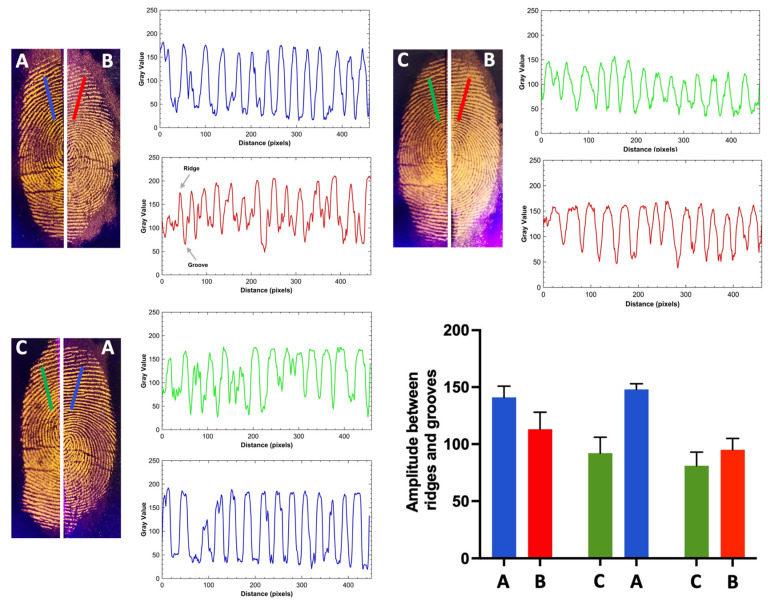
Images of fingerprints developed on aluminum foil with the proposed developers, using the split mode, under UV light (λ = 365 nm) (A: m-DCC@SIL; B: m-DCC@CHT; C: m-DCC@CIT). The Gray Value spectrum and the bar graph of amplitude (difference in intensity between ridges and furrows) of the regular and the magnetic developers are also shown.

Figure 8 shows a comparison between fingerprints developed on aluminum foil surfaces using our regular and magnetic powders based on tri-sodium citrate, chitosan, and silica. For this purpose, fingerprints were deposited on the surface and split into two halves, with each half treated with one of the developers. This approach eliminates intra-donor variability as a potential source of perceived differences between the two techniques or developers applied [40]. To the naked eye, there was no significant difference between the developers, showing very similar results in both color contrast and adhesion. With all the developers, it was possible to discern details of the fingerprint minutiae. However, based on the intensity amplitude between the ridges and furrows observed in the Gray Value spectrum, it is notable that the silica matrix-based developer (m-DCC@SIL) exhibited slightly better contrast with the substrates, particularly in the fine lines or wrinkles present on the tested surface.

### 2.8. Comparison Between Proposed and Commercial Fingerprint Developers

**Figure 9:**
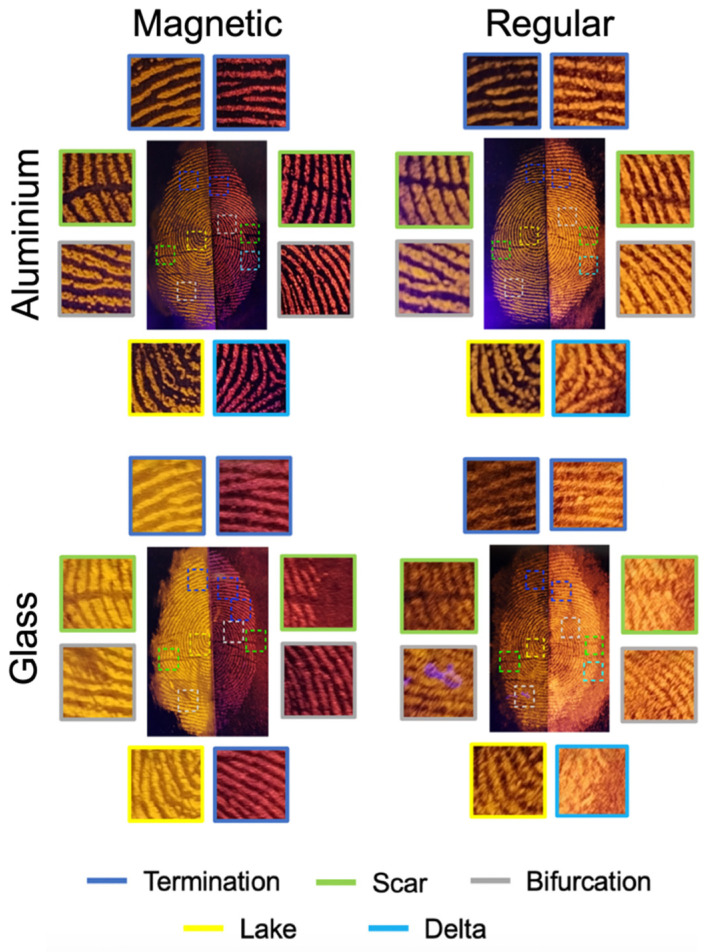
Comparison between fingerprints revealed with the magnetic and regular proposed powders (**left half**) versus magnetic and regular commercial powders (**right half**) by the slipt mode. The fingerprints were deposited on aluminum foil and glass surfaces photographed under UV light (λ = 365 nm).

Figure 9 presents a comparison of fingerprints developed using the proposed magnetic (m-DCC@SIL) and regular (DCC@SIL) powders versus those developed with a commercial reagent (bvda^®^, magnetic fluor red and fluor orange powders) on aluminum foil and glass. To accurately assess the efficiency and sensitivity of our proposed powders, a comparison was conducted in slip mode, enabling a direct scale-based assessment of ridge quality, sensitivity, smudging, and contrast with the background.

Overall, no significant differences were observed between the magnetic and the regular powders on aluminum foil, with both showing comparable color contrast and adhesion to the fingerprints. On glass surface, the proposed magnetic powders displayed slightly better detail and/or contrast than the commercial alternative, whereas the regular powders showed no marked difference in fingerprint detection. In all cases, fingerprints minutiae were clearly discernible.

This result is quantified in Table 2, which provides a comparative analysis based on the absolute scale developed by McLaren et al. [41] This scale allows for a direct visual comparison of split fingerprint halves by assigning scores according to disclosure quality, including detail and/or contrast, relative to the control developer (commercial powders).

## 3. Materials and Methods

### 3.1. Materials

Reagents and solvents were purchased from Sigma-Aldrich (St. Louis, MO, USA) and used as received. 7-(Diethylamino)-2-oxo-2H-chromene-3-carboxylic acid (DCC) was synthesized using a previously reported protocol [42]. The matrices based on chitosan (ChemLab), tri-sodium citrate (Merck, Darmstadt, Germany) and silica (Scharlau, Barcelona, Spain) were pre-treated in a planetary ball mill (Retsch PM100, Verder Group, Haan, Germany) using a zirconium oxide reactor and zirconium oxide balls, to reduce the particle size. Iron (III) chloride hexahydrate (Riedel-de Haën, Honeywell, Charlotte, NC, USA) and iron (II) sulfate hydrate (Merck) were used in the synthesis of Fe_3_O_4_ nanoparticles.

### 3.2. Methods

The fluorescence emission measurements in solid state were determined using a Fluorolog 3 spectrometer (Horiba Jobin Yvon, Longjumeau, France) equipped with a front face accessory for solid samples and the emission spectra were recorded using front face geometry, under ambient temperature (25 °C), with samples sandwiched between quartz slides. The excitation wavelength beam was adjusted to the maximum absorbance. To visualize the fingerprints an ultraviolet (UV) light of 365 nm was used. The magnetic moment of the powders was obtained at room temperature using a magnetometer model EZ7-VSM (MicroSense, Lowell, MA, USA) with a noise level lower than of 1 × 10^−6^ emu. The mass of the quartz cup filled with the powders was measured to enable the calculation of the mass magnetization (emu/g) of the powder samples. The quartz cup was weighted before and after filled with the powders, and the diamagnetic contribution removed. The morphology of the developed powders was analyzed using a Thermo Scientific desktop scanning electron microscope (SEM), model Phenom ProX G6 (Thermo Fisher Scientific model, Waltham, MA, USA), with a CsB6 filament and equipped with a light element energy dispersive, operating at 10 kV at different magnification levels. The samples of each microparticle type were attached with double-sided adhesive tape fixed to carbon stubs, metalized with a thin layer of gold and further examined.

### 3.3. Pre-Treatment and Preparation of Fluorescent Magnetic Powders

The matrices were pre-treated in a planetary ball mill (RETSCH PM100) using a 50 mL zirconium oxide reactor and 5 mm zirconium oxide balls under solvent-free conditions to reduce particle size, operating at 600 rpm for 10 min. For the preparation of the fluorescent magnetic powders (m-DCC@CIT, m-DCC@CHT and m-DCC@SIL) a two-step process was employed: the first step at 350 rpm and the second at 500 rpm, both for 10 min.

### 3.4. Fingerprint Samples

Groomed fingerprint samples were imprinted on paperboard, glass bottle, aluminum and polypropylene surfaces by the same donor. The fingerprint detection was conducted using a magnetic fingerprint brush bvda^®^, B-60000 regular (BVDA International BV, Haarlem, Netherlands), observed under a UV light (λ = 365 nm), and photographed with a Smartphone Camera HONOR, Magic 5 Lite (Honor Device Co., Ltd., Shenzhen, China). The latent fingerprint processed with synthesized powders were compared to those processed with commercial powders using a comparative scale and a split mode technique. This approach minimized variability as a source of perceived differences when two fingerprints are deposited separately. In this method, latent fingerprints were deposited under the same conditions and divided into two halves, with each half revealed with the newly synthesized and commercial powders.

## 4. Conclusions

The synthesized powders demonstrate substantial efficacy for fingerprint detection on various surfaces, offering a sustainable alternative to traditional commercial powders. These powders, developed using a solvent-free ball-milling-assisted coating approach with silica, chitosan, and tri-sodium citrate matrices, maintain strong photophysical properties suitable for forensic applications. Despite a reduction in fluorescence for the magnetic powders due to the presence of Fe_3_O_4_, the intensity remains sufficient for effective use, with chitosan- and tri-sodium citrate-based powders offering enhanced performance due to minimized quenching effects. Additionally, the powders exhibit superparamagnetic behavior, with coated powders showing slightly reduced saturation magnetization, which is expected and acceptable within the application scope.

The comparative analysis indicates that the proposed powders are as effective as commercial options in ridge quality, sensitivity, and contrast, with even improved fingerprint detail on glass surfaces for the magnetic powders. This confirms the potential of these sustainable powders for fingerprint detection, combining environmental benefits with excellent performance. An important advantage of this approach is the use of biodegradable matrices in the formulation of fluorescent magnetic powders, which ensures a sustainable and environmentally friendly alternative for forensic applications.

## Figures and Tables

**Table 1:** Magnetic properties (magnetization, remanence, and coercivity) of non-fluorescent and fluorescent magnetic powders.

Compounds	Ms (emu/g)	Mr (Oe)	Hc (emu/g)	Mr/Ms
Fe_3_O_4_	52.9	0.56	3.53	0.01
m-CHT	3.8	0.05	2.54	0.01
m-CIT	3.4	0.05	6.99	0.02
m-SIL	1.6	0.06	8.73	0.04
m-DCC@CHT	3.3	0.1	4.60	0.03
m-DCC@CIT	1.2	0.02	9.05	0.02
m-DCC@SIL	0.5	0.02	17.33	0.05

Ms = saturation magnetization; Mr = remanent magnetization; Hc = coercive field.

**Table 2:** Relative performance of fingerprint revealed with magnetic proposed powders and commercial powders on aluminum foil and glass surfaces.

Method	Developer	Surface Type	
		Aluminum Foil	Glass
Proposed (half left)	m-DCC@CIT	0	+1
Commercial (half left)	Red Magnetic	0	−1
Proposed (half left)	DCC@CIT	0	0
Commercial (half left)	Orange Flu	0	0

Table key: (+1) commercial developer exhibits slightly greater fingerprint ridge detail and/or contrast than the proposed developer; (0) No significant difference between the commercial and our developer; (−1) commercial developer exhibits slightly lower fingerprint ridge detail and/or contrast than our developer [40,41].

## Data Availability

The original contributions presented in this study are included in the article. Further inquiries can be directed to the corresponding author.
